# Collapsing glomerulopathy in a patient of Indian descent in the setting of COVID-19

**DOI:** 10.1080/0886022X.2020.1811122

**Published:** 2020-08-30

**Authors:** Sarika Deshmukh, Xin Jin Zhou, Wesley Hiser

**Affiliations:** Hill Country Healthcare PLLC, Cedar Park, TX, USA; Renal Path Diagnostics, Pathologists BioMedical Laboratories/PathGroup, Dallas, Texas, USA

Dear Editor,

Coronavirus disease 2019 (COVID-19), caused by the novel coronavirus severe acute respiratory syndrome coronavirus 2 (SARS-CoV-2), was first reported in Wuhan, Hubei Province, China, and has since spread around the globe affecting over 20 million people with a death toll surpassing 700,000. The disease has a variable clinical presentation, ranging from asymptomatic to severe pneumonia and respiratory failure. Many symptomatic patients present with an influenza-like syndrome; however, involvement of other organs including the kidneys has been reported [1, 2]. Although the prevalence of acute kidney injury appears to be lower with COVID-19 compared to that seen in other coronavirus infections [2], one study showed that 44% of patients hospitalized for COVID-19 had evidence of proteinuria at admission, while 27% had hematuria, and 14% had elevated creatinine, all of which were independent risk factors for in-hospital death [3]. While the precise etiology of renal injury in COVID-19 is incompletely understood, tubular injury secondary to virus-induced cytokines, sepsis, and hypoxia, as well as rhabdomyolysis in some cases, has been postulated [1,3]. Additionally, there is evidence to suggest that injury may also be related to direct viral cytotoxicity. Viral-like particles have been detected in tubular epithelial cells and podocytes by electron microscopy, with corresponding positive staining for SARS-CoV nucleoprotein by immunofluorescence [1]. In addition, collapsing glomerulopathy (CG) has recently been reported in several patients with COVID-19 in the United States [4–7]. Interestingly, all cases occurred in patients with high-risk *APOL1* genotypes, which are associated with increased susceptibility to CG and chronic kidney disease.

We report on a 42-year-old man who immigrated to the United States in 2019 from India and developed CG associated with COVID-19. The patient had no significant past medical history, but presented to his primary care physician in January 2020 with fever, cough, and shortness of breath. He received three courses of antibiotics over the next month, but subsequently developed hypertension and lower extremity edema. Upon referral to nephrology in March, he was afebrile but reported a 10 kg weight gain. Laboratory studies revealed a normal serum creatinine (1 mg/dL) with nephrotic-range proteinuria (urine protein-creatinine ratio 8 g/g) and hematuria by urinalysis. He was additionally found to have hypoalbuminemia, hypercholesterolemia, and leukopenia without anemia or thrombocytopenia (Table 1). Serologic studies were negative for hepatitis B and C, HIV, double-stranded DNA, and ANA. He had normal complement C3 and C4, and serum protein electrophoreses was unremarkable. The patient was started on lisinopril 10 mg and a renal biopsy was ordered. Per protocol, the patient underwent COVID-19 testing by nasal swab prior to the procedure, and was found to be positive. Repeat testing two weeks later by polymerase chain reaction (PCR) was again positive. The biopsy was finally performed following a negative test ten days later.

**Table 1.** Select Laboratory Results.

Laboratory test	Patient value	Reference range
Creatinine	1	0.55−1.02 mg/dL
Blood urea nitrogen (BUN)	10	7–25 mg/dL
Albumin	1.9	3.4–5 g/dL
Cholesterol, total	329	0–200 mg/dL
LDL	239	0–100 mg/dL
HDL	59	40–130 mg/dL
Calcium	7.5	8.5–10.1 mg/dL
WBC count	2.8	4.5–11 K/µL
Hemoglobin	15.6	12–16 g/dL
Platelet count	207	140–440 K/µL
Spot urine protein/creatine ratio	8	0–0.1 g/g

**Figure 1.**
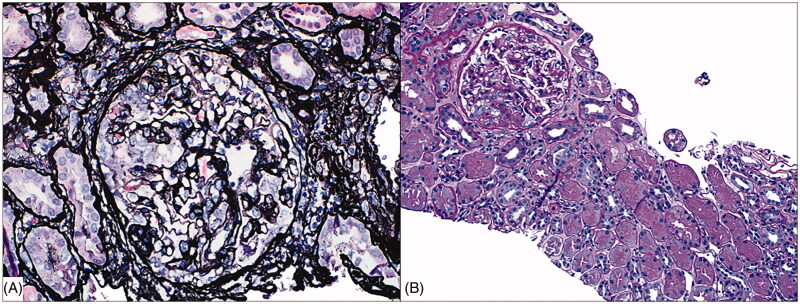
Light microscopy. (A) Glomerulus with segmental collapse of the glomerular tuft with associated hypertrophy and hyperplasia of overlying epithelial cells (Jones methenamine silver, original magnification x400). (B) Glomerulus with segmental sclerosis and collapsing features. There is minimal tubular injury and no significant interstitial inflammation (Periodic acid-Schiff, original magnification x200).

**Figure 2.**
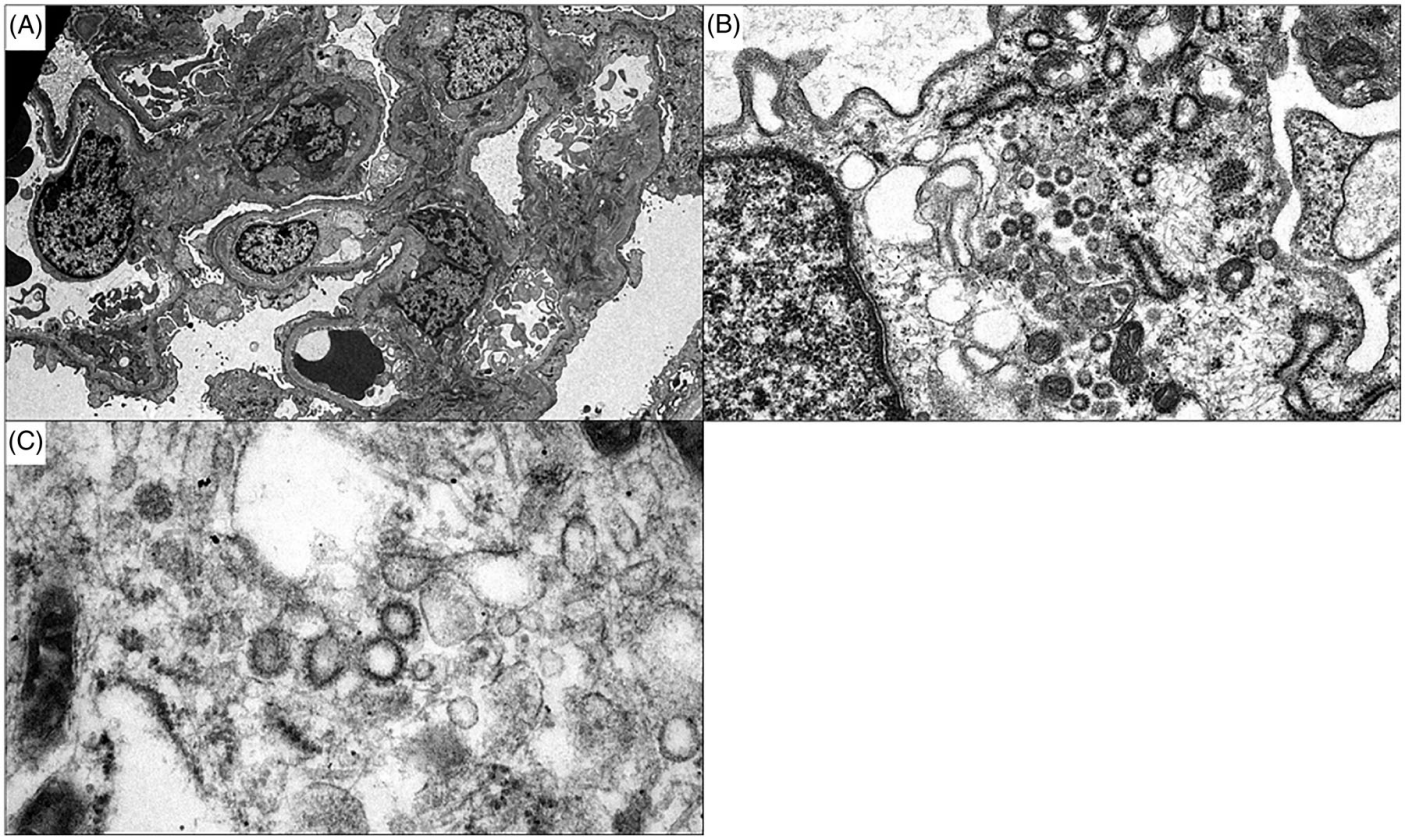
Electron microscopy. (A) Ultrastructural studies show complete foot process effacement of podocytes. Glomerular basement membranes are not significantly thickened and there are no immune complex deposits (original magnification × 2900). (B) Scattered spherical viral-like particles within podocyte cytoplasm with electron-dense rim and protruding spiked projections (original magnification × 30000). (C) Spiked projections protruding from spherical particles are readily visualized at high magnification (original magnification × 68000).

By light microscopy, several glomeruli demonstrated segmental collapse of the capillary tuft with hyperplasia of overlying epithelial cells, some of which contained prominent protein droplets (Figure 1). The cortex showed focal acute tubular injury with minimal interstitial inflammatory infiltrates. There was mild interstitial fibrosis and tubular atrophy with moderate arteriosclerosis. Immunofluorescence studies were negative. Ultrastructural studies showed segmental collapse of the glomerular tuft with numerous enlarged podocytes. Glomerular basement membranes were of normal thickness and texture. There were no immune complex deposits or tubuloreticular inclusions. Podocyte foot processes showed complete effacement. Additionally, numerous spherical particles, measuring between 49 and 102 nm in diameter, were identified within the cytoplasm of podocytes. The particles exhibited an electron dense outer rim and were surrounded by spikes measuring 14–19 nm in length (Figure 2). These particles were similar to previously described viral-like particles in COVID-19 [1], as well as those identified in cases of Middle East respiratory syndrome coronavirus (MERS-CoV) infection [8]. The biopsy was diagnosed as collapsing glomerulopathy, and the presence of viral-like particles within podocytes was suspicious for COVID-19-associated collapsing glomerulopathy. Following the biopsy, infectious disease specialists were consulted; however, the patient did not fit the criteria for emergency use of remdesivir, and was subsequently lost to follow up. Further studies to confirm the presence of SARS-CoV-2 within the biopsy were not performed.

Collapsing glomerulopathy is a unique variant of focal segmental glomerulosclerosis (FSGS) characterized by collapse of the glomerular tuft with hypertrophy and hyperplasia of overlying epithelial cells, often with prominent protein droplets. Most cases have associated acute tubular injury or interstitial nephritis, and some may show microcystic tubular dilation. In addition to primary (idiopathic) FSGS, CG is associated with a number of secondary etiologies. These include infection (e.g., HIV, parvovirus B19, cytomegalovirus, and Epstein-Barr virus), as well as autoimmune diseases including systemic lupus erythematosus, various malignancies, severe ischemia such as that seen with thrombotic microangiopathy, and drugs like interferon and pamidronate [9]. Certain genotypes also play a role in the development of CG, particularly the presence of *APOL1* risk alleles. These *APOL1* variants confer an increased resistance to trypanosomal infection and are found only in individuals of African ancestry, with up to 13% of African-Americans harboring two *APOL1* risk alleles [10]. Patients with high-risk genotypes are at increased risk of chronic kidney disease and CG, and the majority of patients with CG possess an underlying *APOL1* high-risk genotype [4,5]. The precise mechanism through which the podocytes are affected in the setting of *APOL1*-associated CG has not been completely elucidated; however, in many cases cytokine-induced injury is thought to play a major role. In addition, viruses such as HIV and parvovirus B19 may also directly target cells within the kidney, resulting in podocyte injury and contributing to the development of CG [5].

Of the previously reported cases of COVID-19-associated CG, viral-like particles were identified within podocyte vacuoles of one patient; however, PCR performed on frozen tissue for SARS-CoV-2 was negative [6]. The remaining cases demonstrated no direct evidence of viral infection, although tubuloreticular inclusions were identified in one case [4,5,7]. All patients were found to possess *APOL1* high-risk genotypes. Additionally, the previously reported patients all required hospitalization for pulmonary and renal dysfunction. In contrast, our patient had a relatively mild clinical course, not requiring hospitalization, and compared to the other cases, the biopsy showed less exuberant collapsing lesions with milder tubular injury. There were no tubuloreticular inclusions by electron microscopy, but viral-like particles consistent with coronavirus in size and appearance were readily identified within podocytes [1,8]. Serologic workup for underlying autoimmune and infectious etiologies was negative, including repeat HIV testing following the biopsy. Importantly, as a recent immigrant from India, the patient is highly unlikely to possess a high-risk *APOL1* genotype. While additional testing was not performed, the presence of viral-like particles within podocytes in this patient who otherwise carries no risk factors for CG raises suspicion for direct viral toxicity of podocytes.

To our knowledge, this represents the first case of COVID-19-associated CG in a patient with no predisposing risk factors for CG. While the presence of *APOL1* high-risk genotypes likely contributes to the development of CG in many COVID-19 patients, presence of viral-like particles in this case raises the possibility that the virus plays a more significant role in the development of CG than previously indicated, either through direct viral toxicity or cytokine-induced injury. Further investigation into the development of CG in the setting of COVID-19 is critical in order to gain a better understanding of patient outcomes and determine optimal management for affected individuals.
